# The Impact of the COVID-19 Pandemic on Resident Physicians Well-Being in the Surgical and Primary Care Specialties in the United States and Canada

**DOI:** 10.7759/cureus.19677

**Published:** 2021-11-17

**Authors:** Saman Farr, James A Berry, Daniel K Berry, Dario A Marotta, Sara E Buckley, Rida Javaid, Danisi M Jacqueline, Caitlyn E Magargee, Lisa M Ferrouge, Anna M Rogalska, Sepehr Farr, Maria Ahmad, Paras Savla, Dan E Miulli

**Affiliations:** 1 Neurosurgery, Riverside University Health System Medical Center, Moreno Valley, USA; 2 Flight Surgery, Federal Aviation Administration, Kansas City, USA; 3 Department of Research, Alabama College of Osteopathic Medicine, Dothan, USA; 4 Department of Neurology, University of Alabama, Birmingham, USA; 5 Division of Neuropsychology, University of Alabama, Birmingham, USA; 6 School of Osteopathic Medicine, University of the Incarnate Word School of Osteopathic Medicine, San Antonio, USA; 7 Family Medicine, The Donald and Barbara Zucker School of Medicine, Northwell Health-Peconic Bay Medical Center, Riverhead, USA; 8 Psychology, University of La Verne, La Verne, USA; 9 Family Medicine, University of Texas Health Science Center at San Antonio, San Antonio, USA; 10 Family Medicine, Liberty University College of Osteopathic Medicine, Lynchburg, USA; 11 Neurosurgery, Arrowhead Regional Medical Center, Colton, USA

**Keywords:** primary care resident wellness, surgical resident wellness, spiritual well-being, emotional well-being, social well-being, spiritual wellness, resident physician wellbeing, emotional wellness, social wellness, covid-19 pandemic

## Abstract

Purpose: The COVID-19 pandemic disrupted the professional, social, and spiritual activities of resident physicians around the world, impacting wellness and personal relationships. Moreover, social distancing caused significant limitations or shutdown of places of worship, including churches, synagogues, mosques, etc. Our goal was to survey resident physicians in primary care and surgical subspecialties in the United States (U.S.) and Canada and to examine the effect of the COVID-19 pandemic on their well-being.

Methods: An international cross-sectional study was performed in November 2020, using an anonymous survey of programs in the U.S. and Canada, containing 20 questions to assess the impact of the pandemic on resident participation in social and spiritual activities and the effects on their wellness, and personal relationships. The emails with survey links attached were sent to individual program coordinators from accredited residency training programs in the United States and Canada. This consisted of programs accredited by the American Osteopathic Association (AOA), The Royal College of Physicians and Surgeons of Canada (RCPSC), and the Accreditation Council of Graduate Medical Education (ACGME). The survey was evenly divided among surgical programs (General Surgery, Neurological Surgery, Orthopedic Surgery, Urological Surgery, and Integrated Surgical Residency Programs such as Plastic Surgery, Cardiothoracic Surgery, Pediatric Surgery, and Vascular Surgery) as well as primary care programs (Internal Medicine and Family Medicine).

Results: A total of 196 residents, 60 primary care residents, and 136 surgery residents participated in the study. Ninety-six participants (49%) were female, and 98 of the participants (50%) were male, with the remainder two residents identifying as “Other.” Of the primary care residents, the majority (39, 65%) were female. Conversely, the majority (77, 57%) of surgery residents were male.

Conclusion: The COVID-19 pandemic has affected the social lives, relationships, and spiritual well-being of both surgical and primary care resident physicians. However, primary care residents reported significantly greater engagement in personal relationships and were more likely to express feelings of mental and physical exhaustion, prohibiting social attendance.

## Introduction

Focus on residency wellness has increased in recent years. However, after the start of the β-coronavirus 2019-nCoV (COVID-19) pandemic in Wuhan, China [1,2], and the subsequent spread of the virus around the world, social relations were changed worldwide. This has exacerbated the issue of resident physician wellness in the United States and around the world. A major factor contributing to these social changes has been policies such as ‘social distancing’ in an attempt to slow down and limit the spread of the virus. These policies have also resulted in the shutting down or significant limitation of attendance at places of worship, including churches, synagogues, and mosques. While some of this access has been replaced with live streaming or recordings with the implementation of new technologies, physical attendance and interactions have been significantly limited.

At the same time, scarcity of quality information regarding this novel virus, in terms of its transmission, incubation period, the number of infected individuals, geographic extent, morbidity, and mortality rates, etc., has led to significant insecurity and fear among the population [3]. It is well established in the literature that spirituality is a common means through which individuals cope with illness [4]. In fact, up to 65% of patients with depression, anxiety, and other psychiatric conditions indicate that they want spirituality to play a part in their treatment [5]. While there are some studies on the effects of the COVID-19 pandemic on the spirituality of patients and their families, much less is understood regarding its effects on resident physicians (who are often on the frontlines of caring for these patients).

These factors also have a clear effect on resident wellness. One can find numerous studies and literature reviews that evaluate the rates of burnout and its consequences. With the ongoing COVID-19 pandemic, there has been a drastic shift in graduate medical education for numerous reasons, including the need for social distancing as well as deploying residents to cover medical floors with COVID-19 patients. These shifts are hypothesized to have drastic impacts on resident wellness. The impacts of these changes may be felt differently depending on whether the resident is in a surgical specialty or primary care specialty, based on variations in training requirements, time spent at the hospital, and the culture of wellness in the specialty. Few studies have been done on the implications of COVID-19 on resident wellness because it is a novel topic. 

The earliest studies that examined the effect of the COVID-19 pandemic came out of China and observed the effects on the general population. But such studies did not focus exclusively on resident physicians who were actively training in a graduate medical education program [6]. Additionally, political and social biases must always be taken into consideration with regards to publications originating in China, particularly on the topic of the pandemic. Therefore, it is important to have additional peer-reviewed research to help establish the literature in regards to this topic. 

Furthermore, the various components of resident wellness need to be evaluated in the United States because cultural differences between Chinese and American resident physicians can surely affect these populations differently. Several international studies have focused on the structural function of the residency program itself without focusing on the emotional well-being, faith, spirituality, and social lives of the resident physicians [7]. Therefore, the goal of our study was to highlight these aspects of wellness that have largely been ignored by the body of established literature.

## Materials and methods

Participants

A cross-sectional multi-institutional international survey study was conducted across all Accreditation Council for Graduate Medical Education (ACGME), American Osteopathic Association (AOA), and Royal College of Physicians and Surgeons (RCPSC) accredited surgical residency and fellowship programs in the United States and Canada. Surgical residents with an educational status of post-graduate year two (PGY-2) or higher were invited to take part in this study. Program coordinators were sent electronic requests soliciting resident study participation. Electronic communications included instructions and a hyperlink to complete the online survey. This study was approved by the Arrowhead Regional Medical Center Institutional Review Board. Individual consent was implied through survey completion.

Survey

The survey consisted of a 20-question electronic survey using an online computer program (Microsoft Office Forms, registered to the Microsoft Corporation (Redmond, WA). A list of the survey questions is displayed in Table 1 by category. Dichotomous (yes or no) and five-point Likert-scale survey items (strongly disagree, disagree, neutral, agree, and strongly agree) were formulated to assess the impact of COVID-19 on resident social and emotional well-being and the utility of faith during this challenging time. Eight questions assessed social life and personal relationships, six questions assessed impacts and utilities of spiritual well-being, and four questions assessed emotional well-being.

**Table 1:** List of survey questions by category

#	Category	Question Text
Q1	Demographic	Are you currently in a surgical or primary care residency program?
Q2	Demographic	What gender do you identify with?
Q3	Social	Was the last person with whom you were in a relationship during residency, someone you met in your hospital or outside of your hospital?
Q4	Social	In the six months that FOLLOWED the implementation of COVID-19 restrictions, approximately how many days of dating have you been on?
Q5	Social	In the six months PRIOR TO the implementation of COVID-19 restrictions, approximately how many days of dating had you been on?
Q6	Social	How long has it been since you were in a romantic relationship?
Q7	Social	Do you often feel too mentally and physically exhausted to participate in social situations where you could meet a potential dating partner?
Q8	Social	Do you feel that your social life and dating have improved or worsened since you started residency?
Q9	Social	Do you believe that a factor contributing to the end of your last relationship was the burden of your duties and responsibilities as a resident causing a feeling of being physically and/or emotionally absent, or not being able to spend enough time with the person you were dating?
Q10	Social	In light of your duties and responsibilities from your residency program, how would you describe your ability to have an active social life where you could meet someone and begin a relationship?
Q11	Spiritual well-being	My faith makes me feel more secure in life since the start of the COVID-19 pandemic.
Q12	Spiritual well-being	My ability to have meaning in my life through my faith and spirituality has increased since the start of the COVID-19 pandemic.
Q13	Emotional well-being	My ability to deal consciously with others and be empathic has increased since the start of the COVID-19 pandemic.
Q14	Spiritual well-being	My trust in God (or a higher power) has increased since the start of the COVID-19 pandemic.
Q15	Spiritual well-being	My ability to cope with problems through my trust in God (or a higher power) has increased since the start of the COVID-19 pandemic.
Q16	Emotional well-being	My mindfulness (awareness and presence) has increased since the start of the COVID-19 pandemic.
Q17	Emotional well-being	I feel more at peace since the start of the COVID-19 pandemic.
Q18	Emotional well-being	My sense of insight and rationality has increased since the start of the COVID-19 pandemic.
Q19	Spiritual well-being	Faith has an increased role in my decision making since the start of the COVID-19 pandemic.
Q20	Spiritual well-being	My access and ability to attend faith services has increased since the start of the COVID-19 pandemic.

Procedure and data analyses

Survey items were sorted by social, emotional, and faith-based categories. String variables were converted to numeric values. Chi-squared tests were utilized to compare answer choices between groups of residents (surgical versus primary care) and sex differences (male vs. female). We used the phi ($\phi$) coefficient to interpret effect size $\phi = \sqrt{\left ( \frac{\chi ^{2}}{N} \right )}$, where values of .2, .5, and .8 correspond to minimal, moderate, and strong effect sizes, respectively. The data were analyzed in IBM Corp. Released 2020. (IBM SPSS Statistics for Windows, Version 27.0. Armonk, NY: IBM Corp).

## Results

A total of 196 residents, 60 primary care residents, and 136 surgical residents participated in the study. Ninety-six participants (49%) were female, and 98 of the participants (50%) were male, with two participants selecting “Other.” Of the primary care residents, the majority (39, 65%) were female. Conversely, the majority of surgical residents were male (77, 57%). The two participants who did not list their sex were excluded from sex-based comparisons.

Social life

Group comparisons of survey responses related to the impact of COVID-19 on social life and personal relationships are displayed in Table 2. The majority of primary care and surgical residents met their last relationship outside of the hospital, but to a greater degree in the primary care setting, 91.5% vs. 79.7% (p = .042). Similarly, a higher frequency of surgical residents found relationships within the same hospital setting 27 (20%) compared to those in primary care five (9%). These differences were not detected when comparing answers across sexes, where 85% of females and 82% of males found their last relationship outside of the hospital setting.

**Table 2:** COVID-19 and social life: comparison of survey results between surgical and primary care residents

Variable	Residency Type			Sex		
	Primary Care n = 60	Surgery n = 136	χ2 (φ)	Female n = 96	Male n = 98	χ2 (φ)
(Q3) Meeting place of last relationship during residency			4.12 (-.146)*			.276 (.038)
In my hospital	5 (8.5%)	27 (20.3%)		14 (14.9%)	17 (17.7%)	
Outside of my hospital	54 (91.5%)	106 (79.7%)		80 (85.1%)	79 (82.3%)	
(Q4) Number of dates in the last six months FOLLOWING COVID-19			13.59 (.266)**			4.73 (.158)
0	20 (33.9%)	44 (33.1%)		36 (38.3%)	28 (29.2%)	
1-2	15 (25.4%)	13 (9.8%)		14 (14.9%)	14 (14.6%)	
3-4	10 (16.9%)	16 (12.0%)		15 (16.0%)	11 (11.5%)	
5-6	5 (8.5%)	12 (9.0%)		8 (8.5%)	9 (9.4%)	
7 or greater	9 (15.3%)	48 (36.1%)		21 (22.3%)	34 (35.4%)	
(Q5) Number of dates in the last six months PRIOR to COVID-19			17.00 (.297)**			13.25 (.263)**
0	14 (23.7%)	17 (12.7%)		21 (22.1%)	10 (10.4%)	
1-2	13 (22.0%)	10 (7.5%)		16 (16.8%)	7 (7.3%)	
3-4	6 (10.2%)	11 (8.2%)		10 (10.5%)	7 (7.3%)	
5-6	3 (5.1%)	23 (17.2%)		12 (12.6%)	14 (14.6%)	
7 or greater	23 (39.0%)	73 (54.4%)		36 (37.9%)	58 (60.4%)	
(Q6) Time since last romantic relationship			31.53 (.406)***			12.74 (.260)*
Less than 1 month	14 (23.7%)	87 (65.9%)		40 (43.0%)	59 (61.5%)	
1-2 months	18 (30.5%)	5 (3.8%)		7 (7.5%)	6 (6.3%)	
3-4 months	4 (6.8%)	3 (3.8%)		3 (3.2%)	4 (4.3%)	
5-6 months	4 (6.8%)	2 (15.2%)		1 (1.1%)	5 (5.2%)	
Greater than 6 months	29 (49.1%)	35 (26.5%)		42 (45.2%)	22 (22.3%)	
(Q7) Feelings of mental and physical exhaustion prohibit social attendance			4.75 (-.157)*			3.45 (.134)
No	17 (28.8%)	61 (45.5%)		32 (33.7%)	45 (46.9%)	
Yes	42 (71.2%)	73 (54.5%)		63 (66.3%)	51 (53.1%)	
(Q8) Change in social life and dating since the start of residency			.66 (.059)			.48 (.051)
Improved	9 (15.5%)	15 (11.3%)		13 (13.8%)	10 (10.5%)	
Worsened	49 (83.1%)	118 (88.7%)		81 (86.2%)	85 (89.5%)	
(Q9) Residency duties and responsibilities contributed to the end of last relationship			0.23 (.034)			1.16 (.078)
No	26 (44.1%)	64 (47.8%)		48 (51.1%)	42 (43.3%)	
Yes	33 (55.9%)	70 (52.2%)		46 (48.9%)	55 (56.7%)	
(Q10) Ability to have an active social and dating life in light of residency duties and responsibilities			4.15 (.146)			3.93 (.143)
Very difficult	22 (36.7%)	54 (40.0%)		42 (43.8%)	33 (34.0%)	
Somewhat difficult	34 (56.7%)	61 (45.0%)		46 (47.9%)	48 (49.5%)	
Not difficult	3 (5.0%)	11 (8.1%)		5 (5.2%)	9 (9.3%)	
Easy	0 (0.0%)	4 (3.0%)		2 (2.1%)	4 (4.1%)	
Quite easy	1 (1.7%)	5 (3.7%)		1 (1.1%)	3 (3.1%)	
* p-value < 0.05; ** p-value < 0.01; *** p-value < 0.001						

Primary care and surgical residents differed significantly across specialties in terms of the dating frequency when comparing the six months before the pandemic versus after. The majority of surgical residents (73, 54%) reported seven or more dates in the last six months before the start of COVID-19. During the same time, 33 (56%) of primary care residents reported four or lesser dates. This trend nearly reversed following the start of COVID-19. Surgical residents still reported higher frequencies of dating than primary care residents. However, the majority of both primary care residents (45, 77%), and surgical residents (73, 54%), reported less than four dates in the six months following COVID-19. Significant differences in dating frequency did emerge between females and males before COVID-19 (p = .01). The majority of males (68, 60%) reported greater than seven dates compared to females (36, 38%). Twice as many females (21, 22%) reported no dating before COVID-19, compared to males (10, 10%). Differences in dating frequency by sex following COVID-19 were not statistically significant.

Differences in the time since the last romantic relationship were also significant between primary care and surgical resident groups (p < .001) and between females and males (p = .013). Primary care residents reported a quasi-bimodal response distribution, with 32 (54%) and 29 (49%) residents reporting less than two months and greater than six months since their last relationship, respectively. Interestingly, this trend was also evident in female responses, with 40 (43%) and 42 (45%) reporting the same times, respectively, since their last relationship. A majority of surgical residents (87, 66%) and males (59, 62%) report less than one month since their last relationship. A majority of all groups evaluated in this study reported feelings of mental and physical exhaustion during residency, which they felt prohibited attendance at social events. Significant differences existed between the type of resident (p = .029), with 42 primary care residents (71%) being more likely to report these feelings when compared to 73 surgical residents (55%).

The impact of residency duties and responsibilities on social life and personal relationships did not significantly differ by any of the groups evaluated (i.e., primary care, surgical, female, or male). Almost 90% of respondents from each group reported difficulty with having an active social and dating life in light of residency duties and responsibilities. Further, more than 80% of each group reported that their social life and dating frequency worsened since the start of residency. About 50% of each group also reported the duties and responsibilities of residency contributed to the end of their last relationship. Taken together, these findings suggest that the impact of the residency on social and dating lives is ubiquitous and unequivocal among the groups evaluated. Further, the impact of COVID-19 on social life and dating impact residents differently depending on sex and residency type.

Spiritual well-being

Table 3 displays group comparisons of responses to the spiritual well-being survey questions. Overall, significant differences in survey responses were not detected between primary care and surgery resident groups. However, two statistically significant group differences between females and males emerged. First, an increase in ‘trust in God (or a higher power)’ was observed among residents since the start of COVID-19 (p = .046). Second, faith as a mechanism of improved ability to cope with problems since the beginning of the COVID-19 pandemic was also observed among residents (p = .048).

**Table 3:** COVID-19 and spiritual well-being: comparison of survey results between surgical and primary care residents

Variable	Residency Type			Sex		
	Primary Care n = 60	Surgery n = 136	χ2 (φ)	Female n = 96	Male n = 98	χ2 (φ)
(Q11) Faith has contributed to personal feelings of security since COVID-19			1.74 (.095)			.5.56 (.170)
Strongly Disagree	12 (20.0%)	30 (22.2%)		20 (21.1%)	22 (22.4%)	
Disagree	6 (10.0%)	19 (14.1%)		14 (14.7%)	11 (11.2%)	
Neutral	27 (45.0%)	58 (43.0%)		46 (48.4%)	37 (37.8%)	
Agree	11 (18.3%)	17 (12.6%)		9 (9.5%)	19 (19.4%)	
Strongly Agree	4 (6.7%)	11 (8.1%)		6 (6.3%)	9 (9.2%)	
(Q12) Faith has given life meaning since the start of COVID-19			1.45 (.086)			1.44 (.086)
Strongly Disagree	10 (16.7%)	30 (22.0%)		19 (19.8%)	21 (21.4%)	
Disagree	10 (16.7%)	21 (15.4%)		18 (18.8%)	13 (13.3%)	
Neutral	34 (56.7%)	72 (52.9%)		51 (53.1%)	53 (54.1%)	
Agree	4 (6.7%)	6 (4.4%)		4 (4.2%)	6 (6.1%)	
Strongly Agree	2 (3.3%)	7 (5.1%)		4 (4.2%)	5 (5.1%)	
(Q14) Trust in God (or a higher power) has increased since COVID-19			5.88 (.173)			9.66 (.223)*
Strongly Disagree	12 (20.0%)	36 (26.5%)		22 (22.9%)	26 (26.5%)	
Disagree	11 (18.3%)	27 (19.9%)		25 (26.0%)	13 (13.3%)	
Neutral	29 (48.3%)	58 (42.6%)		43 (44.8%)	42 (42.9%)	
Agree	8 (13.3%)	9 (6.6%)		5 (5.2%)	12 (12.2%)	
Strongly Agree	0 (0.0%)	6 (4.4%)		1 (1.0%)	5 (5.1%)	
(Q15) Faith has improved my ability to cope with problems since COVID-19			6.43 (.181)			9.60 (.222)*
Strongly Disagree	12 (20.0%)	35 (25.7%)		21 (21.8%)	26 (26.5%)	
Disagree	12 (20.0%)	27 (19.9%)		27 (28.1%)	12 (12.2%)	
Neutral	27 (45.0%)	59 (43.4%)		39 (40.1%)	45 (45.9%)	
Agree	9 (15.0%)	9 (6.6%)		8 (8.3%)	10 (10.2%)	
Strongly Agree	0 (0.0%)	6 (4.4%)		1 (1.0%)	5 (5.1%)	
(Q19) Faith has an increased role in decision making since COVID-19			2.29 (.108)			4.14 (.146)
Strongly Disagree	16 (26.7%)	46 (34.1%)		28 (29.5%)	34 (34.7%)	
Disagree	19 (31.7%)	37 (27.4%)		31 (32.6%)	24 (24.4%)	
Neutral	20 (33.3%)	42 (31.1%)		31 (32.6%)	30 (30.6%)	
Agree	5 (8.3%)	8 (5.9%)		5 (5.3%)	8 (8.2%)	
Strongly Agree	0 (0.0%)	2 (14.8%)		0 (0.0%)	2 (2.0%)	
(Q20) Access and ability to attend faith services has increased since COVID-19			2.02 (.102)			.43 (.148)
Strongly Disagree	23 (38.3%)	60 (44.4%)		36 (37.9%)	47 (48.0%)	
Disagree	18 (30.0%)	28 (20.7%)		28 (29.5%)	17 (17.3%)	
Neutral	18 (16.9%)	44 (32.6%)		29 (30.5%)	32 (32.7%)	
Agree	1 (8.5%)	3 (2.2%)		2 (2.1%)	2 (2.0%)	
Strongly Agree	0 (0.0%)	0 (0.0%)		0 (0.0%)	0 (0.0%)	
* p-value < 0.05; ** p-value < 0.01; *** p-value < 0.001						

In addition, only six females (6%), when compared to 17 males (17%), reported an ‘increased trust in God (or a higher power)’ since the start of COVID-19. This demographic trend was consistent for the utility of faith as a coping mechanism, but to a lesser degree, with nine females (9%) versus 15 males (15%) in agreement. While the remaining spirituality-based questions did not show significant differences among groups, several trends emerged. Overall, the majority of these responses ranged from disagreement to neutrality. For instance, “neutral” was the most common response in all spirituality-based questions, except ‘faith as a utility for decision making’ in which “strongly disagree” was equally as prevalent. Of particular concern was the observation that the majority of respondents in each group reported reduced access and ability to attend faith services as a result of the COVID-19 pandemic.

Emotional well-being

Table 4 displays group comparisons of responses to emotional well-being survey questions. The vast majority of the respondents indicated a lack of peace since the start of COVID-19. A breakdown of this demonstrated 45 primary care residents (75%) and 100 surgical residents (74%) felt a lack of peace since the start of the pandemic. With respect to gender, 77 females (80%) and 66 males (67%) indicated a lack of peace since the start of COVID-19. Responses to the remaining survey questions evaluating insight and rationality, mindfulness, and empathy indicated somewhat of a normal distribution with a median response centered at a viewpoint of neutrality. There was no overwhelming prevalence indicating positive or negative changes in these domains. Of note, none of the emotional well-being questions were statistically different among the groups that were analyzed.

**Table 4:** COVID-19 and emotional well-being: comparison of survey results between surgical and primary care residents

Variable	Residency Type			Sex		
	Primary Care n = 60	Surgery n = 136	χ2 (φ)	Female n = 96	Male n = 98	χ2 (φ)
(Q13) Empathy has increased since the start of COVID-19			5.26 (.164)			6.04 (.197)
Strongly Disagree	3 (5.0%)	13 (9.6%)		9 (9.4%)	7 (7.1%)	
Disagree	14 (23.3%)	38 (27.9%)		25 (26.0%)	25 (25.5%)	
Neutral	23 (38.3%)	59 (43.4%)		39 (40.6%)	43 (43.9%)	
Agree	18 (30.0%)	23 (16.9%)		23 (24.0%)	18 (18.4%)	
Strongly Agree	2 (3.3%)	3 (2.2%)		0 (0.0%)	5 (5.1%)	
(Q16) Mindfulness (awareness and presence) have increased since the start of COVID-19			2.47 (.113)			6.74 (.187)
Strongly Disagree	3 (5.0%)	16 (30.7%)		8 (8.4%)	11(11.2%)	
Disagree	16 (26.7%)	30 (22.2%)		23 (24.2%)	23 (23.5%)	
Neutral	22 (36.7%)	47 (34.8%)		31 (32.6%)	37 (37.8%)	
Agree	18 (3.0%)	39 (28.9%)		33 (34.7%)	23 (23.5%)	
Strongly Agree	1 (1.7%)	3 (2.2%)		0 (0.0%)	4 (4.1%)	
(Q17) The feeling of peace has increased since COVID-19			4.88 (.158)			8.39 (.209)
Strongly Disagree	29 (48.3%)	50 (37.0%)		46 (48.4%)	32 (32.7%)	
Disagree	16 (26.7%)	50 (37.0%)		31 (32.6%)	34 (34.7%)	
Neutral	7 (11.7%)	24 (17.8%)		9 (9.5%)	22 (22.4%)	
Agree	7 (11.7%)	9 (6.7%)		8 (8.4%)	8 (8.2%)	
Strongly Agree	1 (1.7%)	2 (1.5%)		1 (1.1%)	2 (2.0%)	
(Q18) Insight and rationality has increased since COVID-19			5.61 (.230)			5.94 (.175)
Strongly Disagree	5 (8.3%)	16 (11.9%)		12 (12.6%)	9 (9.2%)	
Disagree	23 (38.3%)	30 (22.2%)		32 (33.7%)	20 (20.4%)	
Neutral	22 (36.7%)	61 (45.1%)		36 (37.9%)	47 (48.0%)	
Agree	9 (15.0%)	24 (17.8%)		13 (13.7%)	19 (19.4%)	
Strongly Agree	1 (1.7%)	4 (2.7%)		2 (2.1%)	3 (3.1%)	
* p-value < 0.05; ** p-value < 0.01; *** p-value < 0.001						

## Discussion

Guidelines for social behavior during the COVID-19 pandemic have included mask usage, distancing between people of at least six feet, and limiting time in public areas, including restaurants, grocery stores, and other indoor spaces such as museums, concerts venues, etc. [8]. These types of restrictions dramatically change options for dating and socialization in light of social distancing and mask usage [9]. Work guidelines included increased distance from other co-workers, Plexiglas partitions, and staggering breaks, so employees are not in the same room during that time [10]. This increase in social distancing has led to a lack of social network that typically provides emotional support to residents. For example, many residents have not met their entire residency cohort. Residents at baseline before the COVID-19 pandemic suffered high rates of anxiety, burnout, and social isolation [11]. The increased isolation from COVID-19 precaution guidelines both at and outside of work, and limitations on social interactions, may further contribute to resident burnout and feelings of social isolation [8,12,13].

Emotional stress during residency has been linked to long-lasting consequences. A prospective study by Raimo et al. has shown that emotional stress in residency may be linked to future emotional stress and burnout, up to 10 years into future practice [14]. The COVID-19 pandemic has been associated with anxiety based on generalized anxiety disorder 7-item scale (GAD-7) scores at all levels of residency training, although it was noted to be disproportionately higher for senior-level residents [15,16]. In addition to changes in the workplace and social interaction, sexual and romantic experiences have also changed due to the COVID-19 pandemic. It is reported in the literature that while there has been an overall decline in the number of sexual interactions, there has been an increase in the use of sex toys, sexually explicitly messaging, and more variations in sexual activities when intercourse occurs [17]. The landscape of dating has also changed. Dating apps before the COVID-19 pandemic were losing the number of users they previously once had. During the pandemic, however, the pendulum has swung back, and global online dating is reported to have increased by a factor of 82 [10]. Some dating platforms have even altered their software to allow for video chat dating [12].

The consequences of burnout and social isolation are huge and can even include suicide, which is of importance since physicians already have a higher rate of suicide than the general population [18]. In addition to suicide, burnout can lead to substance abuse, which can directly impact patient care, well-being, and many other aspects of the individual’s life [19,20]. To date, there has been minimal research evaluating perceived stress levels and burnout among the different specialties.

Overall, both the necessary public health measures and alterations to traditional residency duties during the time of COVID-19 have led to increased anxiety and stress on medical residents. Prior research has shown that elevated stress levels may reach a critical point and have long-term effects on the physician’s future practice, attrition in the medical field, and increased risk of suicide attempts or completions. By evaluating resident wellness regarding the specialty, the graduate medical education community will have a tool that may assist them in taking a more individualized approach to resident wellness and support that is program specific to the needs of its residents. The graduate medical education community must act to not only recognize the triggering factors for increased burdens on the medical residents but also to mitigate excess stress and anxiety, as it will have term and potentially detrimental effects on the medical profession that will be long lasting.

## Conclusions

Overall, social lives and personal relationships were impacted by COVID-19, as well as general residency duties and responsibilities. While surgery residents were more likely to engage in dating activities, both primary care and surgery residents saw a significant reduction in dating frequency and social event attendance as a result of the pandemic. Primary care residents reported significantly greater times since engaging in personal relationships and were more likely to express feelings of mental and physical exhaustion prohibiting social attendance. Before COVID-19, male residents engaged in dating activity more frequently. However, this effect leveled out among the sexes after COVID-19. Regardless of sex or residency type, participants reported residency duties and responsibilities as a barrier to an active social and dating life. With regards to spiritual well-being, most respondents were neutral or disagreed with faith as a contributor to security, giving meaning to life, or having an increased role in decision making since the start of COVID-19. However, spiritual well-being was significantly affected by the pandemic as males reported significantly higher trust in God or a higher power and the utility of faith as a coping mechanism in response to the pandemic. Furthermore, the majority of residents reported reduced access and ability to attend faith services since COVID-19. Lastly, the vast majority of participants reported a reduced sense of peace since the start of COVID-19. Our study suggests that for primary care and surgical resident physicians in the U.S. and Canada, nearly all aspects of social and dating life, as well as spiritual well-being and access to faith services, have been negatively impacted by COVID-19. Further studies into the causality of reduced social interaction before versus after COVID-19 are needed.
